# Parental depressive symptoms link family income to preschool child mental health in Western China

**DOI:** 10.3389/fpubh.2026.1836791

**Published:** 2026-06-17

**Authors:** Hongli Sun, Jia Wang, Mingyue Duan, Tingrui Zhang, Xi Zhang

**Affiliations:** 1 School of Humanities and Social Sciences, Xi'an Jiaotong University, Xi'an, China; 2 Shaanxi Institute for Pediatric Diseases, Xi’an Key Laboratory of Children‘s Health and Diseases, Xi’an Children‘s Hospital (Affiliated Children’s Hospital of Xi‘an Jiaotong University), Xi'an, China; 3 School of Politics, Law & Public Administration, Yan’an University, Yan'an, China; 4 Department of Clinical Laboratory, Xi’an Children‘s Hospital, Affiliated Children’s Hospital of Xi‘an Jiaotong University, National Regional Children’s Medical Center (Northwest), Xi'an, China; 5 School of Economics and Management, Xiamen University – Malaysia, Sepang, Malaysia

**Keywords:** child mental health, family income, SDQ, parental depressive symptoms, Western China

## Abstract

**Background:**

Child mental health represents a global public health priority. However, evidence regarding the association between family income and mental health among preschool children in Western China remains limited, and the potential indirect role of parental depressive symptoms has not been fully examined.

**Methods:**

This cross-sectional study included 21,366 preschool children (aged 3–6 years) and their parents in Western China. Family income was classified into low, middle, and high groups. Child mental health outcomes (total difficulties and prosocial behavior) were assessed using the Strengths and Difficulties Questionnaire (SDQ), and parental depressive symptoms were measured via the Center for Epidemiological Studies Depression Scale (CES-D). Multivariable logistic regression and mediation analyses were performed, adjusting for relevant covariates.

**Results:**

Higher family income was significantly associated with a reduced risk of child mental health problems and increased prosocial behaviors. Compared with low-income families, middle- and high-income families had 27 and 39% lower odds of total difficulties, and 9 and 30% higher odds of prosocial behavior, respectively. Parental depressive symptoms accounted for 30.5 and 44.3% of the associations with total difficulties, and 57.2 and 43.8% of the associations with prosocial behavior, in middle- and high-income groups, respectively.

**Conclusion:**

Family income is associated with preschool mental health in Western China, with parental depressive symptoms playing a substantial indirect role. These findings provide population-level evidence for understanding early childhood mental health in understudied regions of China.

## 1 Introduction

Mental health issues affect 10–20% of children and adolescents worldwide (1). Early psychological and behavioral problems predict long-term educational impairment and psychosocial morbidity, with lifelong consequences (1, 2). The preschool period (ages 3–6 years) represents a critical window for neurodevelopment, emotional regulation, and social skill acquisition, during which mental health problems can have enduring effects on developmental trajectories (3–5). Several unique features distinguish this developmental stage from later childhood and adolescence, warranting dedicated investigation. First, preschool years are characterized by heightened neuroplasticity and rapid maturation of emotion-regulatory circuits, making this period particularly sensitive to environmental influences, especially maternal support and family context (6–8). Second, unlike school-aged children or adolescents who spend substantial time in peer and school environments, where classroom-level adversity and peer dynamics increasingly shape behavioral outcomes (9), preschoolers remain primarily dependent on their family environment, and this dependency places parental mental health and family socioeconomic conditions as the most direct and potent determinants of their well-being (8, 10). Third, while externalizing problems in older children often manifest as observable behaviors such as truancy, classroom disruption, or misconduct (9), early childhood mental health difficulties typically present as internalizing symptoms (e.g., excessive crying, sleep disturbances, irritability) or less visible behavioral dysregulation (10, 11). These subtler manifestations make preschool mental health problems more likely to be overlooked by caregivers and healthcare systems (10). Despite its importance, population-level evidence on early childhood mental health and its modifiable risk factors remains limited in many underdeveloped regions, especially in Western China.

Family socioeconomic context plays a fundamental role in shaping child mental health outcomes. Family income, a core component of socioeconomic status, has been identified as a key social determinant of health (12). Evidence from high-income countries indicates that low family income is linked to increased risk of emotional and behavioral problems in children, through pathways including material deprivation, environmental stressors, and impaired parenting quality (13–15). China’s rapid urbanization has exacerbated income inequality, particularly in Western China where GDP per capita remains the lowest among the nation’s four major regions (16–19). Children in this region face compounded risks, including rural–urban disparities constrained by hukou policies and frequent parental migration (20–22). Although previous studies in Eastern China have reported associations between family income and child mental health (23), the generalizability of these findings is limited due to significant economic, cultural, and structural differences, creating a critical evidence gap in research on the relationship between income and mental health.

Parental mental health is widely recognized as a major proximal determinant of child emotional and behavioral development. Parental depressive symptoms, in particular, have been consistently associated with increased risk of emotional difficulties, behavioral problems, and impaired social functioning in children across early development (24, 25). Depressive symptoms can reduce parental sensitivity, increase irritability, and disrupt supportive parent–child interactions, directly undermining children’s psychological security and adaptive development (26–28). Despite the well-established link between parental depression and child mental health, few population-based studies have explored this relationship among preschool children in resource-limited regions of China.

The Family Stress Model (FSM) provides a theoretical framework suggesting that economic disadvantage may contribute to parental psychological distress, which in turn impairs family functioning and increases child psychosocial risk (29). This pathway has been supported in Western populations and appears especially influential during early childhood (30, 31), yet has been only partially validated among school-aged children in China (32). Parental depressive symptoms may therefore serve as an important intermediate factor linking family income to child mental health. Most previous studies have used binary income comparisons (low vs. high) (33, 34), which may mask potential gradient or non-linear effects across low-, middle-, and high-income groups (35). It remains unclear whether parental depressive symptoms mediate the association between family income gradient and mental health outcomes in preschoolers in Western China.

However, the applicability of the Family Stress Model in Western China warrants careful contextual consideration for several reasons. First, the region’s lower GDP per capita and higher poverty rates create a context where even small absolute increases in family income may have larger marginal benefits for reducing parental psychological distress compared to wealthier regions (16, 18). In other words, the income–parental distress link might be steeper in Western China due to more pronounced material hardship. Second, China’s household registration (hukou) system — a birth-based categorization into agricultural (rural) and non-agricultural (urban) status — ties eligibility for locally funded public services (e.g., public kindergartens, subsidized healthcare) to formal registration rather than actual residence (36). Families with rural hukou who migrate to cities often cannot access local public education or health services without paying substantially higher fees, or may be excluded entirely (16). Meanwhile, high-quality educational and healthcare resources are concentrated in larger cities, while rural and remote areas remain underserved (37). Thus, rural-hukou families face a double disadvantage: when they stay in their registered rural areas, services are limited and lower in quality; when they migrate for better economic opportunities, they are eligible for urban public services only after local registered residents have been served, often leaving them with limited residual access unless they pay high out-of-pocket costs (38). Consequently, many parents with rural hukou are forced to leave their children behind in the registered rural areas while they work in cities, causing both parents and children to face long-term parent–child separation, reduced caregiving quality, and increased vulnerability to mental health problems (21, 22). These institutional and geographical barriers may partially limit the extent to which increased family income can be translated into improved child health and educational resources for rural-hukou families. Third, extended family structures and multigenerational child rearing remain common in China, which could modify or buffer the effects of parental depression on child outcomes compared to the nuclear family contexts in Western countries (39). Collectively, these unique socioeconomic and cultural features suggest that the FSM’s core pathway — from economic hardship through parental distress to child maladjustment — may operate with different strength or salience in Western China. Empirical validation of this model in this understudied population is therefore overdue.

This population based cross-sectional study aimed to: (1) examine associations between family income (low, middle, high) and preschool child mental health outcomes (total difficulties and prosocial behavior); and (2) investigate the indirect role of parental depressive symptoms in these associations.

## 2 Method

### 2.1 Study design and participants

This secondary analysis utilized cross-sectional data from a large-scale epidemiological survey conducted among preschool children and their primary caregivers, employing a stratified multistage cluster sampling strategy to ensure population representativeness. The sampling framework was designed as follows: First, all 13 administrative regions in Yan’an City, Shaanxi Province, Western China (including municipal districts and counties) were selected to cover diverse socioeconomic contexts (urban/rural, developed/developing areas). Yan’an is a representative prefecture-level city in Western China, with a per capita GDP and urban–rural structure at the mid-tier level among western regions, making it broadly comparable to other non-provincial-capital and non-border areas in Western China. Secondly, 189 public kindergartens were randomly selected from the official registration list, and the sample size was allocated according to the proportion of children in each region. Finally, all children aged 3–6 years in the selected kindergartens were invited to participate, with one primary caregiver (defined as the family member mainly responsible for the child’s routine daily care and companionship, a qualitative definition widely used in pediatric mental health and population-based epidemiological studies (40, 41)) completing the questionnaire.

Data collection occurred between February 28 and March 5, 2025. All questionnaires were completed by parents using paper forms distributed by trained kindergarten teachers. This mode of administration avoided potential selection bias that could arise from online surveys, which might exclude families with limited internet access. The original survey initially recruited 25,017 caregiver-child dyads to explore determinants of early childhood mental health. The present study investigated the association between family income and preschool child mental health. Building on this foundation, we further developed examined the mediating role of parental depressive symptoms in this association, and therefore we excluded 2,408 non-parent caregivers. Missing data were limited exclusively to covariates (missing or implausible age values: parental age, *n* = 517; child age, *n* = 726), with complete records available for all core study variables. After excluding missing covariate data, the final analytical sample comprised 21,366 parental caregiver-child dyads.

Primary parental caregivers were selected as informants for three reasons: (1) they have the most frequent daily interactions with children, enabling valid assessment of child mental health; (2) as both the direct earners of family income and the individuals most proximally affected by family economic conditions, they are uniquely positioned to reflect the pathway linking family income to child outcomes through their own mental health; and (3) this approach maintains consistency with the original survey design. Data were collected via primary parent-reported questionnaires, including the Strengths and Difficulties Questionnaire (SDQ) and Center for Epidemiologic Studies Depression Scale (CES-D).

The study received ethical approval from Medical Ethics Committee (Approval No. 20250225-21). All procedures followed Declaration of Helsinki principles, with anonymized data and guardian consent. This study followed the Strengthening the Reporting of Observational Studies in Epidemiology (STROBE) guideline for cross-sectional studies.

### 2.2 Measurements

#### 2.2.1 Child mental health outcomes

The official simplified Chinese version of the SDQ is utilized to assess mental health outcomes. This tool comprises 25 items and has demonstrated reliability and validity among Chinese children aged 3 to 17 (42, 43). This study primarily employs the SDQ to evaluate two specific domains: total difficulties and prosocial behavior. The total difficulties subscale showed acceptable internal consistency (Cronbach’s *α* = 0.703), while the prosocial behavior subscale demonstrated good internal consistency (Cronbach’s *α* = 0.833). This level of reliability is consistent with prior Chinese validation studies of the SDQ in preschool populations (44, 45). For analysis, both child mental health outcomes were treated as binary variables, as detailed below.

(1) Total Difficulties Score (TDS): A composite measure (range: 0–40) derived from four subscales including emotional symptoms, conduct problems, hyperactivity/inattention, and peer problems, and each scored on a 3-point Likert scale (0=“does not apply” to 2 = “completely applies”), with five items (7, 11, 14, 21, 25) reverse-scored. A total difficulties score above 14 is defined as being at risk of mental health problems, and this cut-off represents a clinically meaningful threshold validated in previous studies (46).

(2) Prosocial behavior: A specialized scale (range: 0–10) indicates that higher scores reflect stronger intentions for prosocial behavior, with a score below 6 representing a potential deficiency. This cut-off of <6 is a clinically established threshold (46).

#### 2.2.2 Parental depressive symptoms

Parental depressive symptoms were assessed using the CES-D, a validated tool for depression screening in community samples (47). This 20-item scale evaluates symptom frequency (depressed mood, fatigue, appetite loss, and sleep disturbances) over the preceding week using a 4-point scale. Total scores range 0–60, with higher scores indicating greater symptom frequency. The measure demonstrates strong internal consistency (present sample Cronbach’s *α* = 0.849) and has the same validity in China (48) and in Western countries (49).

#### 2.2.3 Family income

In the surveys, the respondents were asked to report annual family income choosing from a series of eight predefined income bands. To create three meaningful income groups for analysis, we referred to China’s National Bureau of Statistics report, which classifies residents into five income brackets, with middle quintile: 32,195 CNY (approximately USD 4,508) and high quintile: 95,055 CNY (approximately USD 13,310) (19, 20). Guided by these reference values, the middle bracket (32,195 CNY) and the high bracket (95,055 CNY) were rounded to 30,000 CNY (approximately USD 4,201) and 100,000 CNY (approximately USD 14,002), respectively. Further combining the predefined questionnaire income intervals and the actual income distribution of our study sample, annual family income was stratified into three categories: Low-income group (less than 30,000 CNY/4,201 USD), Middle-income group (30,000 to 99,999 CNY/4,201 to 14,001 USD), and High-income group (100,000 CNY/14,002 USD or more). The final cut-offs were selected because they aligned well with the predefined income bands in the questionnaire and ensured sufficient sample sizes in each group for robust statistical analysis, while capturing extreme income disparities.

#### 2.2.4 Covariables

Based on the established association with children’s mental health outcomes, the following covariates were included in the analyses: Core demographics (child gender/age, primary parent gender/age), family structure (number of children), healthy behaviors (smoking/alcohol use), marital status, parental education level, parental occupation, household registration, and early education participation.

### 2.3 Statistical analysis

Three nested models were constructed to progressively account for confounding factors: Model 1 was unadjusted. Model 2 was adjusted for core demographics (child gender/age, primary parent gender/age). Model 3 further incorporated factors that affect resource allocation and parenting quality, including family structure (number of children), healthy behaviors (smoking/alcohol use), marital status, parental education level (50–52), parental occupation, household registration, and early education participation (53, 54). This stratified approach sequentially controls proximal (demographic/behavioral) to distal (socioeconomic) confounders, while accounting for layered socioeconomic influences on child development.

To ensure the robustness of the regression model, we used the variance inflation factor (VIF) statistic to diagnose multicollinearity among independent variables and covariates. All VIF values ranged from 1.0 to 2.4, well below the commonly accepted threshold of 5, indicating that there was no serious multicollinearity among the covariates. Therefore, all variables were retained in the subsequent regression models (Supplementary Table S1).

Continuous variables were reported as mean ± standard deviation (SD) and categorical variables as frequencies (*n*, %). We categorized the baseline characteristics based on the children’s mental health outcomes (total difficulties score and prosocial behavior score) for subsequent analyses. Between-group comparisons used the Kruskal–Wallis test for continuous variables and Fisher’s exact test for categorical variables with expected counts <10 were used, to compare differences in baseline characteristics across the three income groups. We categorized baseline characteristics by income tier (low/middle/high).

Multivariable logistic regression was used to assess: (1) the association between family income and child mental health outcomes (total difficulties and prosocial behavior), and (2) the association between parental depressive symptoms and child mental health outcomes, with additional adjustment for family income in the fully adjusted model to isolate the independent effect of the mediator. Multivariable linear regression examined the association between family income and parental depressive symptoms. All regression models were sequentially adjusted for demographic, socioeconomic, and family covariates. Stratified analyses and interaction tests examined effect modification across subgroups.

Mediation analyses were performed to examine the indirect role of parental depressive symptoms in the association between family income and child mental health outcomes. Given the cross-sectional design, the observed indirect role should be interpreted as a statistical association rather than a causal pathway. Since family income was treated as a three-category variable (low, middle, high), mediation analyses were conducted separately for the middle- versus low-income and high- versus low-income comparisons, with the low-income group serving as the reference. We first adjusted for parental depressive symptoms in regression analyses to observe changes in child mental health outcomes before and after adjustment. And then the indirect effect was estimated using regression-based mediation analysis, adjusting for child characteristics, primary caregiver characteristics, and family socioeconomic factors, and was further verified by bootstrap resampling with 1,000 iterations to calculate bias-corrected 95% confidence intervals. Statistical significance was determined when the confidence interval excluded zero. The proportion mediated was calculated to quantify the magnitude of the indirect effect. Following the method proposed by Fritz and Mackinnon (55), a post-hoc power analysis was conducted for the mediation model. Given the large sample size (*N* = 21,366) and the observed magnitude of the indirect effect, the statistical power for detecting the mediation effect was greater than 0.80, indicating sufficient power to identify the indirect associations in this study.

For the sensitivity analysis, we retained the full sample of 22,609 parent–child dyads (after excluding non-parental caregivers but before excluding the 1,243 age-missing records), while all main analyses were performed on the complete-case sample of 21,366 dyads. In this full sample, missing data were observed for certain covariates, including parental age (*n* = 517, 2.29%) and child age (*n* = 726, 3.21%). Assuming a missing at random (MAR) mechanism, multiple imputation by chained equations (MICE) with five imputed datasets was applied to handle missing values, incorporating family income, child mental health outcomes, parental depressive symptoms, and all demographic and socioeconomic covariates as predictor variables (56, 57). Results from the multiple imputation analysis were compared with those from the complete-case analysis to assess robustness. Analyses were performed using R software (v4.3.2; R Foundation) and EmpowerStats (X&Y Solutions), with statistical significance defined as *p* < 0.05 (two-tailed).

## 3 Results

### 3.1 Demographic

Table 1 presents the baseline characteristics of 21,366 participants stratified by child mental health outcomes (total difficulties and prosocial behavior). Children with a total difficulties score >14 exhibited significantly higher parental depressive symptoms (CES-D: 15.25 ± 7.60 vs. 10.27 ± 6.00), a higher proportion of low-income families (49.45% vs. 38.26%), and a lower proportion of high-income families (14.08% vs. 20.52%) relative to those with a score ≤14. They were also more likely to have male primary caregivers, younger parental age, lower parental education levels, higher rates of parental smoking and alcohol use, rural household registration, and non-intact family status (all *p* < 0.001). For prosocial behavior, children with a score <6 showed significantly higher parental depressive symptoms (12.29 ± 6.63 vs. 10.13 ± 6.44), a higher proportion of low-income families (42.15% vs. 38.57%), male primary caregivers, parental smoking and alcohol use, and lower rates of urban household registration (all *p* < 0.05). Baseline characteristics stratified by annual family income are provided in Supplementary Table S2.

**Table 1:** Characteristics of participants by child mental health outcomes (*N* = 21,366).

Characteristics	Total difficulties			Prosocial behavior		
	Normal, ≤14 (*N* = 17,388)	At risk of mental health problems, >14 (*N* = 3,978)	*p-*value	Potential prosocial deficiency, <6 (*N* = 10,558)	Normal, ≥6 (*N* = 10,108)	*p-*value
Child gender			<0.001			<0.001
Boys	8,897 (51.17%)	2,165 (54.42%)		5,712 (54.10%)	5,350 (49.50%)	
Girls	8,491 (48.83%)	1813 (45.58%)		4,846 (45.90%)	5,458 (50.50%)	
Child age, years	4.82 ± 0.88	4.80 ± 0.90	0.256	4.74 ± 0.91	4.89 ± 0.86	<0.001
Primary parent gender			<0.001			<0.001
Male	3,961 (22.78%)	1,147 (28.83%)		2,792 (26.44%)	2,316 (21.43%)	
Female	13,427 (77.22%)	2,831 (71.17%)		7,766 (73.56%)	8,492 (78.57%)	
Primary parent age, years	34.81 ± 4.53	34.48 ± 4.68	<0.001	34.65 ± 4.52	34.85 ± 4.61	0.001
CES-D	10.27 ± 6.00	15.25 ± 7.60	<0.001	12.29 ± 6.63	10.13 ± 6.44	<0.001
Annual family income			<0.001			<0.001
Low-income	6,652 (38.26%)	1967 (49.45%)		4,450 (42.15%)	4,169 (38.57%)	
Middle-income	7,168 (41.22%)	1,451 (36.48%)		4,247 (40.23%)	4,372 (40.45%)	
High-income	3,568 (20.52%)	560 (14.08%)		1861 (17.63%)	2,267 (20.98%)	
Number of children			0.004			0.366
1	4,981 (28.65%)	1,167 (29.34%)		2,994 (28.36%)	3,154 (29.18%)	
2	10,527 (60.54%)	2,317 (58.25%)		6,395 (60.57%)	6,449 (59.67%)	
≥3	1880 (10.81%)	494 (12.42%)		1,169 (11.07%)	1,205 (11.15%)	
Education level			<0.001			<0.001
≤Junior high school	4,337 (24.94%)	1,404 (35.29%)		3,001 (28.42%)	2,740 (25.35%)	
High school diploma	3,353 (19.28%)	740 (18.60%)		1982 (18.77%)	2,111 (19.53%)	
Junior college	4,447 (25.58%)	936 (23.53%)		2,664 (25.23%)	2,719 (25.16%)	
≥Undergraduate degree	5,251 (30.20%)	898 (22.57%)		2,911 (27.57%)	3,238 (29.96%)	
Occupation			<0.001			<0.001
Education/medical workers	2,487 (14.30%)	430 (10.81%)		1,321 (12.51%)	1,596 (14.77%)	
Enterprise managers/government civil servants/technicians	3,224 (18.54%)	642 (16.14%)		1921 (18.19%)	1945 (18.00%)	
Sales staff/service workers /freelancers	4,482 (25.78%)	973 (24.46%)		2,671 (25.30%)	2,784 (25.76%)	
Housewife/husband	4,110 (23.64%)	1,108 (27.85%)		2,598 (24.61%)	2,620 (24.24%)	
Unemployment/retirement/others	3,085 (17.74%)	825 (20.74%)		2047 (19.39%)	1863 (17.24%)	
Marital status			<0.001			0.084
Married/cohabitating	16,939 (97.42%)	3,822 (96.08%)		10,280 (97.37%)	10,481 (96.97%)	
Widowed/divorced/separated	449 (2.58%)	156 (3.92%)		278 (2.63%)	327 (3.03%)	
Smoking status			<0.001			<0.001
Yes	2,436 (14.01%)	743 (18.68%)		1753 (16.60%)	1,426 (13.19%)	
No	14,952 (85.99%)	3,235 (81.32%)		8,805 (83.40%)	9,382 (86.81%)	
Alcohol status			<0.001			<0.001
Yes	2,670 (15.36%)	838 (21.07%)		1951 (18.48%)	1,557 (14.41%)	
No	14,718 (84.64%)	3,140 (78.93%)		8,607 (81.52%)	9,251 (85.59%)	
Household registration of children			<0.001			0.013
Urban	5,993 (34.47%)	1,123 (28.23%)		3,431 (32.50%)	3,685 (34.10%)	
Rural	11,395 (65.53%)	2,855 (71.77%)		7,127 (67.50%)	7,123 (65.90%)	
Attended early education program			0.630			<0.001
Yes	5,585 (32.12%)	1,262 (31.72%)		3,232 (30.61%)	3,615 (33.45%)	
No	11,803 (67.88%)	2,716 (68.28%)		7,326 (69.39%)	7,193 (66.55%)	

Continuous variables are expressed as mean±SD. Categorical variables are expressed as frequency (*n*, %).

### 3.2 Univariate associations between variables and mental health outcomes in preschool children

Univariate associations between variables and child mental health outcomes (total difficulties and prosocial behavior) are summarized in Supplementary Table S3. Parental depressive symptoms (CES-D score) were strongly correlated with both outcomes: each unit increase in CES-D was associated with 11% higher odds of total difficulties (OR = 1.11, 95% CI [1.10, 1.12], *p* < 0.001) and 5% lower odds of prosocial behavior (OR = 0.95, 95% CI [0.94, 0.95], *p* < 0.001). A clear income gradient emerged: compared to the low-income group, children in middle- and high-income households had 32 and 47% lower odds of total difficulties, respectively, and 10 and 30% higher odds of prosocial behavior (all *p <* 0.001). Sociodemographic characteristics also showed notable associations. Girls had lower odds of total difficulties but higher odds of prosocial behavior relative to boys. Additionally, having the mother as the primary caregiver (OR = 0.73, 95% CI [0.67, 0.79], *p <* 0.001) and higher parental education (≥undergraduate degree: OR = 0.53, 95% CI [0.48, 0.58], *p <* 0.001) were associated with lower odds of total difficulties, whereas parental smoking (OR = 1.41, 95% CI [1.29, 1.54], *p <* 0.001) and alcohol use (OR = 1.47, 95% CI [1.35, 1.60], *p <* 0.001) were linked to increased odds of total difficulties. For prosocial behavior, urban household registration (OR = 0.93, 95% CI [0.88, 0.99], *p* = 0.0132) and early education participation (OR = 0.88, 95% CI [0.83, 0.93], *p <* 0.001) were associated with favorable outcomes.

### 3.3 Associations between family income and mental health outcomes in preschool children

Table 2 presents the association between family income and child mental health outcomes across three progressive adjustment models. Compared with the low-income group, middle-income and high-income families were consistently associated with lower odds of total difficulties and higher odds of prosocial behavior in preschool children across all models (all *p* < 0.01). In the fully adjusted Model 3, the protective effect of family income remained significant: the OR for total difficulties was 0.73 (95% CI [0.68, 0.79]) for middle-income and 0.61 (95% CI [0.54, 0.68]) for high-income families, while the OR for prosocial behavior was 1.09 (95% CI [1.03, 1.16]) and 1.30 (95% CI [1.20, 1.41]), respectively. These associations were robust even after stepwise comprehensive covariate adjustment.

**Table 2:** Multivariable logistic regression between family income and mental health outcomes (*N* = 21,366).

Family income	Model 1		Model 2		Model 3	
	OR (95% CI)	*p-*value	OR (95% CI)	*p-*value	OR (95% CI)	*p-*value
Total difficulties						
Low-income	1.0 (ref)		1.0 (ref)		1.0 (ref)	
Middle-income	0.68 [0.63, 0.74]	<0.001	0.68 [0.63, 0.73]	<0.001	0.73 [0.68, 0.79]	<0.001
High-income	0.53 [0.48, 0.59]	<0.001	0.52 [0.47, 0.57]	<0.001	0.61 [0.54, 0.68]	<0.001
Prosocial behavior						
Low-income	1.0 (ref)		1.0 (ref)		1.0 (ref)	
Middle-income	1.10 [1.04, 1.17]	0.0020	1.12 [1.05, 1.19]	0.0003	1.09 [1.03, 1.16]	0.0049
High-income	1.30 [1.21, 1.40]	<0.001	1.36 [1.26, 1.46]	<0.001	1.30 [1.20, 1.41]	<0.001

Model 1: Crude model without adjustment. Model 2: Adjusted for child gender, child age, primary parent gender, primary parent age. Model 3: Model 2 plus number of children, smoke, alcohol use, marital status, education level, occupation, household registration of children and early education.

### 3.4 Association between family income and parental depressive symptoms

Linear regression analysis confirmed a robust inverse association between family income gradient and parental depressive symptoms (Table 3). Compared with the low-income group, middle- and high-income parents exhibited significantly lower CES-D scores across all models. In the fully adjusted model (Model 3), middle-income status was associated with a 0.94-point reduction in parental depressive symptoms (*β* = −0.94, 95% CI [−1.13, −0.74], *p <* 0.001), while high-income status was associated with a 2.18-point reduction (*β* = −2.18, 95% CI [−2.43, −1.92], *p <* 0.001), indicating a clear dose–response gradient.

**Table 3:** Multivariable linear regression between family income and parental depressive symptoms (CES-D score) (*N* = 21,366).

Family income	Model 1		Model 2		Model 3	
	*β* (95% CI)	*p-*value	*β* (95% CI)	*p-*value	*β* (95% CI)	*p-*value
Low-income	0 (ref)		0 (ref)		0 (ref)	
Middle-income	−1.31 [−1.51, −1.12]	<0.001	−1.30 [−1.50, −1.11]	<0.001	−0.94 [−1.13, −0.74]	<0.001
High-income	−2.93 [−3.18, −2.69]	<0.001	−2.92 [−3.17, −2.68]	<0.001	−2.18 [−2.43, −1.92]	<0.001

Model 1: Crude model without adjustment. Model 2: Adjusted for child gender, child age, primary parent gender, primary parent age. Model 3: Model 2 plus number of children, smoke, alcohol use, marital status, education level, occupation, household registration of children and early education. β, unstandardized regression coefficient; CI, confidence interval.

### 3.5 Associations between parental depressive symptoms and mental health outcomes in preschool children

Table 4 presents the association between parental depressive symptoms and child mental health outcomes across three progressive adjustment models. In the unadjusted model (Model 1), each unit increase in CES-D score was associated with 11% higher odds of total difficulties (OR = 1.11, 95% CI [1.10, 1.12]) and 5% lower odds of prosocial behavior (OR = 0.95, 95% CI [0.94, 0.95]). Adjusting for child and parent demographics (Model 2) did not alter these estimates. Critically, after further adjustment for family income and all other covariates in Model 3, the associations remained statistically significant and stable (total difficulties: OR = 1.11, 95% CI [1.10, 1.11]; prosocial behavior: OR = 0.95, 95% CI [0.94, 0.95]; both *p <* 0.001), indicating that the relationship between parental depressive symptoms and child mental health outcomes is independent of family income and other sociodemographic factors.

**Table 4:** Multivariable logistic regression between parental depressive symptoms and mental health outcomes (*N* = 21,366).

Outcome	Model 1		Model 2		Model 3	
	OR (95% CI)	*p-*value	OR (95% CI)	*p-*value	OR (95% CI)	*p-*value
Total difficulties	1.11 [1.10, 1.12]	<0.001	1.11 [1.10, 1.12]	<0.001	1.11 [1.10, 1.11]	<0.001
Prosocial behavior	0.95 [0.94, 0.95]	<0.001	0.95 [0.94, 0.95]	<0.001	0.95 [0.94, 0.95]	<0.001

Model 1: Crude model without adjustment. Model 2: Adjusted for child gender, child age, primary parent gender, primary parent age. Model 3: Model 2 plus number of children, smoke, alcohol use, marital status, education level, occupation, household registration of children and early education.

### 3.6 The indirect effect through parental depressive symptoms

**Figure 1:**
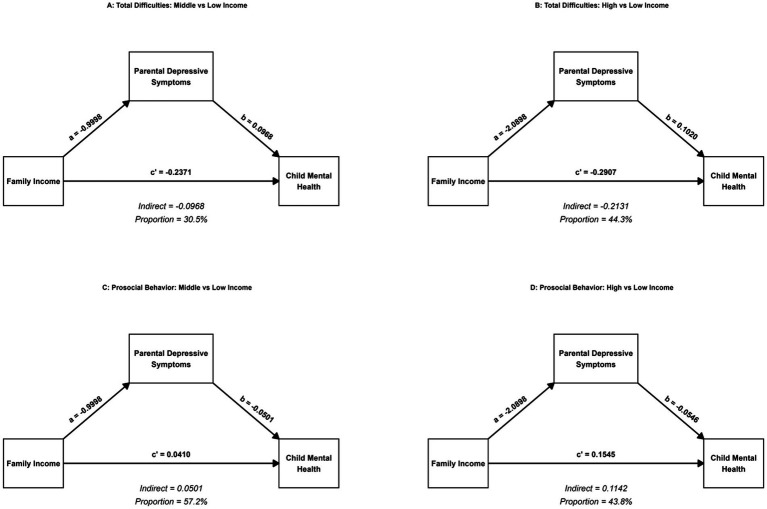
Mediation effects of parental depressive symptoms on the association between family income and child mental health outcomes (*N* = 21,366) **(A)** total difficulties (Middle- vs. Low-income), **(B)** total difficulties (High- vs. Low-income), **(C)** prosocial behavior (Middle- vs. Low-income), and **(D)** prosocial behavior (High- vs. Low-income).

Table 5 presents mediation analyses examining the indirect role of parental depressive symptoms in the association between family income and child mental health outcomes. After adjusting for CES-D scores, the protective associations of higher income attenuated, with ORs for total difficulties increasing from 0.68 to 0.76 (middle-income) and from 0.53 to 0.69 (high-income). Parental depressive symptoms accounted for 30.46 and 44.32% of the association with total difficulties, respectively. For prosocial behavior, the ORs decreased from 1.10 to 1.03 (middle-income) and from 1.30 to 1.13 (high-income) after CES-D adjustment, with mediated proportions of 57.16 and 43.80%. These findings indicate that parental depressive symptoms explain a substantial portion of the income-related disparities in child mental health outcomes, as illustrated in the path diagram (Figure 1). Detailed estimates of path a, path b, path c′, total, direct, indirect effect, and mediated proportions are presented in Supplementary Tables S4, S5.

**Table 5:** Mediation analyses of parental depressive symptoms between family income and mental health outcomes (*N* = 21,366).

Family income	Unadjusted		Adjusted with CES-D		Mediation proportion (%) (95%CI)	*p* value
	OR(95%CI)	*p-*value	OR(95%CI)	*p-*value		
Total difficulties						
Low-income	1.0 (ref)		1.0 (ref)			
Middle-income	0.68 [0.63, 0.74]	<0.001	0.76 [0.71, 0.83]	<0.001	30.46% [23.56, 40.24%]	<0.001
High-income	0.53 [0.48, 0.59]	<0.001	0.69 [0.62, 0.77]	<0.001	44.32% [36.09, 58.30%]	<0.001
Prosocial behavior						
Low-income	1.0 (ref)		1.0 (ref)			
Middle-income	1.10 [1.04, 1.17]	0.0020	1.03 [0.97, 1.10]	0.3242	57.16% [32.39, 85.16%]	<0.001
High-income	1.30 [1.21, 1.40]	<0.001	1.13 [1.04, 1.22]	0.0021	43.80% [36.66, 67.49%]	<0.001

### 3.7 Stratification and interaction analyses

**Figure 2:**
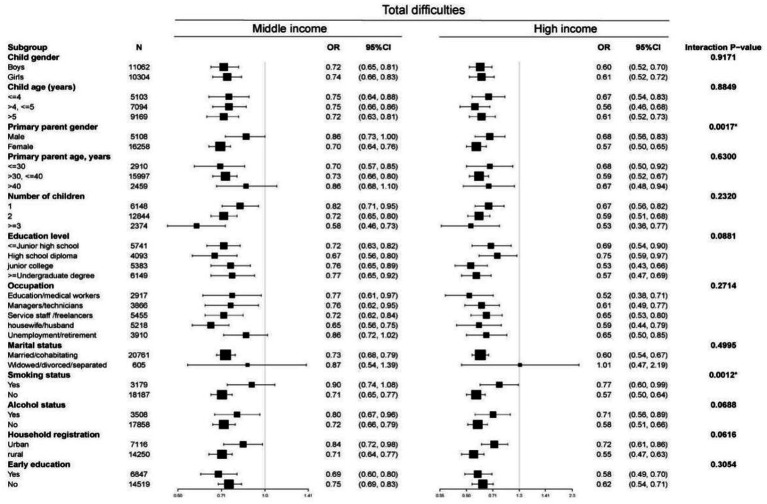
Forest plots of the association between family income gradient and total difficulties in various subgroups. Low income as reference; *p < 0.01.

**Figure 3:**
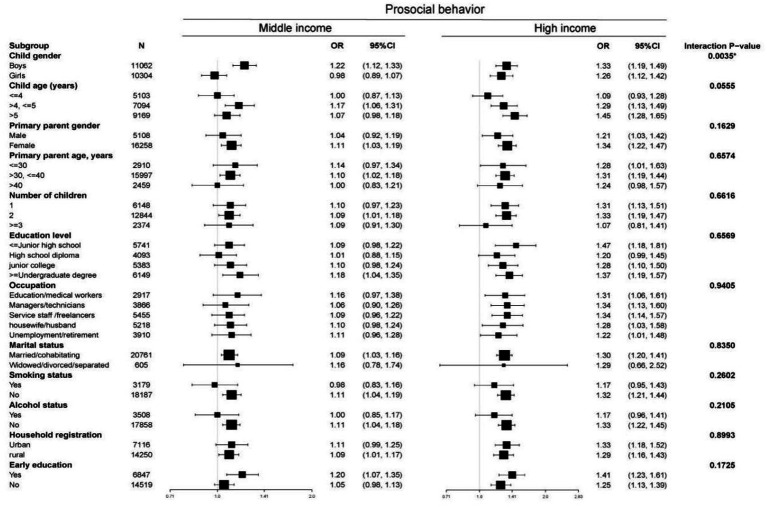
Forest plots of the association between family income gradient and prosocial behavior in various subgroups. Low income as reference; *p < 0.01.

Stratified analyses and interaction tests were performed to examine potential effect modification across subgroups. For total difficulties, the inverse association with higher income remained stable across most subgroups (all *p*-interaction >0.05). Significant effect modification was observed only for primary parent gender (*p*-interaction = 0.0017) and household smoking status (*p*-interaction = 0.0012). The magnitude of the association was weaker in families with male caregivers (middle-income: OR = 0.86, 95% CI [0.73, 1.00]; high-income: OR = 0.68, 95% CI [0.56, 0.83]) than those with female caregivers (middle-income: OR = 0.70, 95% CI [0.64, 0.76]; high-income: OR = 0.57, 95% CI [0.50, 0.65]), and weaker in smoking households (middle-income: OR = 0.90, 95% CI [0.74, 1.08]; high-income: OR = 0.77, 95% CI [0.60, 0.99]) than nonsmoking households (middle-income: OR = 0.71, 95% CI [0.65, 0.77]; high-income: OR = 0.57, 95% CI [0.50, 0.64]) (Figure 2). For prosocial behavior, the positive association with higher income demonstrated consistent patterns across most subgroups, with significant modification by child gender only (*p* for interaction = 0.0035). The income gradient was more pronounced in boys (middle-income: OR = 1.22, 95% CI [1.12, 1.33]; high-income: OR = 1.33, 95% CI [1.19, 1.49]) than in girls, who showed non-significant associations in middle-income families (OR = 0.98, 95% CI [0.89, 1.07]) despite comparable high-income benefits (OR = 1.26, 95% CI [1.12, 1.42]) (Figure 3).

To address missing data, sensitivity analyses were conducted using multiple imputation. After combining the regression coefficients and standard errors from five sets of regression models, the results were consistent with the main analysis, supporting the robustness of our study findings (Supplementary Table S6).

## 4 Discussion

This cross-sectional study of 21,366 preschool children in Western China revealed three principal findings. First, family income was strongly associated with preschool children’s mental health:compared to low-income peers, children in middle- and high-income households had 27 and 39% lower odds of total difficulties, and 9 and 30% higher odds of prosocial behavior, respectively, after full covariate adjustment. Second, parental depressive symptoms demonstrated strong independent associations with both outcomes, with each unit increase in CES-D score corresponding to 11% higher odds of total difficulties and 5% lower odds of prosocial behavior, even after adjusting for family income. Third, mediation analyses revealed that parental depressive symptoms accounted for substantial proportions of the family income–mental health associations: 30.5 and 44.3% of the protective effects for total difficulties in middle- and high-income groups, respectively, and 57.2 and 43.8% of the associations with prosocial behavior. This suggests that parental depressive symptoms play an important indirect role in the association between family income and children’s mental health outcomes.

Our data show that after adjusting for relevant factors, a higher family income gradient is associated with a reduced risk of potential mental health problems in children, which is consistent with prior evidence. Importantly, the protective influence of socioeconomic advantage is particularly pronounced during developmentally sensitive periods, such as early childhood and pre-adolescence (58). The consistency of our results with this developmental principle further supports why the preschool stage examined in this study is a critical window for understanding income-related mental health disparities. Family income determines the material resources available to households (59), including nutritional status, housing conditions, access to health services and educational opportunities-all of which are closely related to child mental health (15). Gallegos et al.’s (60) systematic review confirmed that food insecurity, a common consequence of low income, impairs children’s psychosocial functioning, while Hair et al. (61) documented that chronic economic deprivation leads to altered gray matter development in low-income children, with long-term neurocognitive and mental health impacts. Additionally, housing quality, community safety, and environmental conditions, which vary with socioeconomic status, further mediate the association between income and child mental health (62), as economic instability limits access to quality care and increases exposure to adverse childhood experiences (63). Conversely, higher-income families typically inhabit resource-rich environments with superior nurturing and early education access, enhancing cognitive stimulation and social–emotional development (64).

We use mediation analysis to explore whether and to what extent parental depressive symptoms play an indirect role in the association between family income and child mental health outcomes. After adjusting for CES-D, the protective effect of high income is attenuated, and the mediation analysis shows that parental depressive symptoms account for 30.5–57.2% of the association between family income and mental health. The mediating role of parental depressive symptoms identified in this study provides empirical support for the Family Stress Model (29) in the Chinese context and aligns with growing evidence that parental mental health serves as a critical conduit through which economic conditions affect children. Financial constraints may heighten psychological stress, reduce social support, and limit access to preventive health services, all of which contribute to the onset or exacerbation of parental depressive symptoms (65, 66). Parental depressive symptoms, in turn, hinder effective parenting practices, reducing positive emotional interactions and cognitive stimulation that are essential for preschool children’s mental health development (67, 68). Prior research has linked parental depression to impaired parent–child interactions and worsened internalizing and externalizing outcomes (69, 70), with quasi-experimental evidence demonstrating that maternal depression and economic hardship are tightly interlinked and transmit risk to children through negative parenting practices (71). Law et al. (72) identified perinatal maternal depression as a mediator of income-related deficits in school readiness. Additionally, children of depressed low-income mothers exhibit poorer adaptive skills and more emotional and behavioral problems than socioeconomic-matched controls (73), highlighting that parental depression amplifies the negative impact of low income on child mental health. Our findings extend this evidence by quantifying, for the first time in a Western China preschool population, the proportion of income-related mental health disparities attributable to parental depressive symptoms, with mediated proportions approaching 50% in several comparisons. Collectively, while material deprivation directly harms child mental health, psychological sequelae, especially parental depression, tend to make the negative effects of low income on children’s mental health more severe or even long-lasting (34, 74).

Our stratified analysis revealed potential interaction effects that further clarify the role of parental and family factors in the association between income and child mental health. The protective effects of higher income were not uniform across all subgroups. For total difficulties, significant effect modification emerged for primary parent gender and household smoking status. The income gradient was attenuated in families where the father is the primary caregiver and in smoking households. Regarding father caregiver families, this pattern may be due to maternal employment providing a second household income, thereby alleviating economic difficulties. It may also be explained by inherent differences in parenting styles: paternal primary care tends to provide more flexible or relaxed daily routines, greater participation in outdoor activities, and lower economic-related parenting stress, which can buffer mental health differences between children from low- and high-income families and weaken the income gradient effect (75). Furthermore, emerging evidence suggests that father involvement serves as a protective factor for maternal mental health (76); greater paternal engagement in childcare and household chores has been associated with lower maternal depressive symptoms, which in turn benefits child development and overall family well-being. This indirect pathway — father caregiving alleviating maternal distress — may also contribute to the buffering effect of father primary caregiving on the income-child mental health gradient. Regarding smoking households, the blunted income benefit is likely not driven by tobacco exposure alone; instead, household smoking may act as a proxy for more complex family stressors, including lower socioeconomic resilience, unhealthy lifestyle clustering, and elevated familial psychological strain (77). These underlying household adversities may offset and dilute the protective mental health effects of higher family income, resulting in a muted income gradient among smoking families. These findings suggest that adverse family dynamics-including tobacco exposure and gender-differentiated caregiving patterns-may diminish the mental health benefits typically associated with higher income. For prosocial behavior, significant modification by child gender was observed: the income gradient was more pronounced in boys, while girls showed non-significant associations in middle-income families despite comparable high-income benefits. This pattern may reflect son preference and gender-differentiated parenting practices prevalent in some Chinese cultural contexts (78, 79), wherein families allocate more resources and attention to boys’ social development. These interaction findings underscore the importance of considering family-level contextual factors when examining income–mental health relationships. Salimiha et al. (80) also demonstrated that maternal employment and income levels are closely intertwined, exerting mixed impacts on children’s socio-emotional outcomes, while low income increases ADHD risk through the mediation of family dynamics (81). Furthermore, the COVID-19 pandemic confirmed that financial stress disrupts family dynamics, increases harsh parenting, and negatively affects both parental and child mental health (82), which aligns with our observation that adverse family factors interact with income to influence child mental health.

Our findings suggest that parental depressive symptoms represent a modifiable intervention target for public health programs. This finding provides empirical support for integrating parental mental health into early childhood public health strategies, offering a potential pathway to mitigate income-related disparities in child mental health outcomes in Western China.

### 4.1 Strengths and limitations

Our research has three strengths. First, stratified cluster sampling of 21,366 preschool children in 189 kindergartens in Western China ensured geographical/demographic diversity and enhanced generality to the context of rapidly urbanizing low- and middle-income countries. Second, the mediation analysis quantified the indirect effects of parental depression, providing evidence of an indirect association between parental depressive symptoms and the mental health of preschool children. Third, income class stratification (low/middle/high) captures nonlinear gradient that transcends binary comparisons. Adjust sequentially for the 12 covariates across hierarchical domains (demographic to socioeconomic).

Our results should take into account a few limitations. Firstly, given the cross-sectional design of this study, the possibility of causal inference is excluded, and a bidirectional relationship (e.g., child mental health → parental depression) cannot be ruled out. Specifically, while our study focuses on the effect of parental depression on children’s mental health, we also acknowledge that children’s behavioral problems may in turn increase parental stress and depressive symptoms, which may affect the interpretation of the observed associations. The cross-sectional design cannot distinguish between these competing directions. Future longitudinal studies are needed to formally test the temporal ordering of these pathways and establish causal precedence. Secondly, there are residual confounding factors that have not been measured, such as genetic risk and neighborhood social capital, which may partially explain these associations. Thirdly, the CES-D only assesses the frequency of symptoms but cannot diagnose clinical depression. Fourthly, all child mental health outcomes were evaluated based on parent-reported questionnaires, which may introduce potential reporting bias. Lastly, existing evidence suggests that the SDQ has relatively limited measurement accuracy in young preschool children, particularly for children aged 3 years (83).

## 5 Conclusion

This cross-sectional study of 21,366 preschool children in Western China provides evidence that higher family income is associated with a reduced risk of child mental health problems and increased prosocial behaviors. Parental depressive symptoms play an important indirect role in these associations, mediating 30.46–57.16% of the links. These findings underscore the importance and influence of parental mental health in early childhood, as well as the potential of alleviating socioeconomic stress to break negative intergenerational cycles in child mental health.

## Data Availability

The data that support the findings of this study are available on request from the corresponding author. The data are not publicly available due to privacy or ethical restrictions. Requests to access these datasets should be directed to Xi Zhang, zhangxi00425@163.com.
