# Supplementary material for: Parental depressive symptoms link family income to preschool child mental health in Western China
Source: Front Public Health. 2026 Jun 17;14:1836791. doi: 10.3389/fpubh.2026.1836791 (PMC13319042; doi:10.3389/fpubh.2026.1836791)
Supplement: Supplementary file 2 [file Table_2.DOCX]

**Supplementary Material: Details of Income Classification**

This supplementary material provides additional information on the measurement and categorization of family income, as referred to in Section 2.2.3 of the main manuscript.

**Part 1** Original survey question on annual family income (eight predefined bands)

Participants were asked to report their total annual family income by selecting one of the following eight categories:

29. What is your average annual family income? [Single choice]

○ Less than 10,000 CNY

○ 10,000 – 29,999 CNY

○ 30,000 – 59,999 CNY

○ 60,000 – 99,999 CNY

○ 100,000 – 149,999 CNY

○ 150,000 – 199,999 CNY

○ 200,000 – 299,999 CNY

○ 300,000 CNY or more

**Part 2** Official disposable income quintiles of Chinese residents (2023, National Bureau of Statistics of China)

The official five income strata based on national resident disposable income are presented as follows:

Low-income group: 9,215 CNY

Lower-middle income group: 20,442 CNY

Middle income group: 32,195 CNY

Upper-middle income group: 50,220 CNY

High-income group: 95,055 CNY
