# Supplementary material for: Parental depressive symptoms link family income to preschool child mental health in Western China
Source: Front Public Health. 2026 Jun 17;14:1836791. doi: 10.3389/fpubh.2026.1836791 (PMC13319042; doi:10.3389/fpubh.2026.1836791)
Supplement: Supplementary file 3 [file Table_3.DOCX]

**Table S1 the VIF values of independent variables and covariates**

| **Variable** | **VIF** |
| --- | --- |
| Total difficulties | 1.1 |
| Prosocial behavior | 1.1 |
| Parental depressive symptoms (CES-D) | 1.1 |
| Annual family income | 1.1 |
| Child gender | 1.0 |
| Child age, years | 1.0 |
| Primaryparent gender | 2.4 |
| Primary parent age, years | 1.2 |
| Number of children | 1.2 |
| Education level | 1.7 |
| Occupation | 1.3 |
| Marital status | 1.0 |
| Smoking status | 2.3 |
| Alcohol status | 1.8 |
| Household registration of children | 1.3 |
| Attended early education program | 1.1 |

VIF = variance inflation factor, calculated as 1/(1-R²). Stepwise screening was applied with a threshold of 5. All variables exhibited VIF < 5, indicating no substantial multicollinearity.

Table S2 Baseline characteristics of the study participants by family income (N=21,366)

| **Variable** | **Total sample** |  | **Annual family income** | | | | ***p*-value** |
| --- | --- | --- | --- | --- | --- | --- | --- |
|  |  |  | **Low-income** | **Middle- income** | | **High-income** |  |
|  | N = 21366 |  | N = 8619 | N =8619 | | N =4128 |  |
|  | Mean±SD/ N (%) |  | Mean±SD/ N (%) | | | |  |
| **Child gender** |  |  |  | |  |  | .875 |
| Boys | 11062 (51.77%) |  | 4457 (51.71%) | | 4453 (51.66%) | 2152 (52.13%) |  |
| Girls | 10304 (48.23%) |  | 4162 (48.29%) | | 4166 (48.34%) | 1976 (47.87%) |  |
| **Child age, years** | 4.82 ± 0.89 |  | 4.85 ± 0.89 | | 4.81 ± 0.88 | 4.76 ± 0.90 | <.001 |
| **Primary parent gender** |  |  |  | |  |  | <.001 |
| Male | 5108 (23.91%) |  | 1874 (21.74%) | | 2025 (23.49%) | 1209 (29.29%) |  |
| Female | 16258 (76.09%) |  | 6745 (78.26%) | | 6594 (76.51%) | 2919 (70.71%) |  |
| **Primary parent age, years** | 34.75 ± 4.56 |  | 34.68 ± 4.76 | | 34.60 ± 4.43 | 35.21 ± 4.37 | < .001 |
| **Number of children** |  |  |  | |  |  | < .001 |
| 1 | 6148 (28.77%) |  | 2134 (24.76%) | | 2579 (29.92%) | 1435 (34.76%) |  |
| 2 | 12844 (60.11%) |  | 5261 (61.04%) | | 5185 (60.16%) | 2398 (58.09%) |  |
| ≥ 3 | 2374 (11.11%) |  | 1224 (14.20%) | | 855 (9.92%) | 295 (7.15%) |  |
| **Education level** |  |  |  | |  |  | < .001 |
| ≤ Junior high school | 5741(26.87%) |  | 3285 (38.11%) | | 2053 (23.82%) | 403 (9.76%) |  |
| High school diploma | 4093(19.16%) |  | 1848 (21.44%) | | 1647 (19.11%) | 598 (14.49%) |  |
| junior college | 5383(25.19%) |  | 1957 (22.71%) | | 2385 (27.67%) | 1041 (25.22%) |  |
| ≥ Undergraduate degree | 6149(28.78%) |  | 1529 (17.74%) | | 2534 (29.40%) | 2086 (50.53%) |  |
| **Occupation** |  |  |  | |  |  | < .001 |
| Education/medical workers | 2917 (13.65%) |  | 893 (10.36%) | | 1347 (15.63%) | 677 (16.40%) |  |
| Enterprise managers/government civil servants/technicians | 3866 (18.09%) |  | 915 (10.62%) | | 1480 (17.17%) | 1471 (35.63%) |  |
| Sales staff/service workers /freelancers | 5455 (25.53%) |  | 2104 (24.41%) | | 2399 (27.83%) | 952 (23.06%) |  |
| housewife/husband | 5218 (24.42%) |  | 2866 (33.25%) | | 1954 (22.67%) | 398 (9.64%) |  |
| Unemployment/retirement/others | 3910 (18.30%) |  | 1841 (21.36%) | | 1439 (16.70%) | 630 (15.26%) |  |
| **Marital status** |  |  |  | |  |  | < .001 |
| Married/cohabitating | 20761 (97.17%) |  | 8240 (95.60%) | | 8440 (97.92%) | 4081 (98.86%) |  |
| Widowed/divorced/separated | 605 (2.83%) |  | 379 (4.40%) | | 179 (2.08%) | 47 (1.14%) |  |
| **Smoking status** |  |  |  | |  |  | < .001 |
| Yes | 3179 (14.88%) |  | 1173 (13.61%) | | 1308 (15.18%) | 698 (16.91%) |  |
| No | 18187 (85.12%) |  | 7446 (86.39%) | | 7311 (84.82%) | 3430 (83.09%) |  |
| **Alcohol status** |  |  |  | |  |  | < .001 |
| Yes | 3508 (16.42%) |  | 1242 (14.41%) | | 1415 (16.42%) | 851 (20.62%) |  |
| No | 17858 (83.58%) |  | 7377 (85.59%) | | 7204 (83.58%) | 3277 (79.38%) |  |
| **Household registration of children** |  |  |  | |  |  | < .001 |
| Urban | 7116 (33.31%) |  | 2098 (23.34%) | | 2830 (32.83%) | 2188 (53.00%) |  |
| rural | 14250 (66.69%) |  | 6521 (75.66%) | | 5789 (67.17%) | 1940 (47.00%) |  |
| **Attended early education program** |  |  |  | |  |  | < .001 |
| Yes | 6847 (32.05%) |  | 2285 (26.51%) | | 2714 (31.49%) | 1848 (44.77%) |  |
| No | 14519 (67.95%) |  | 6334 (73.49%) | | 5905 (68.51%) | 2280 (55.23%) |  |
| **CES-D** | 11.20 ± 6.62 |  | 12.29 ± 6.93 | | 10.98 ± 6.36 | 9.36 ± 6.00 | < .001 |
| **Total difficulties** |  |  |  | |  |  | < .001 |
| ≤14 | 17388 (81.38%) |  | 6652 (77.18%) | | 7168 (83.17%) | 3568 (86.43%) |  |
| >14 | 3978 (18.62%) |  | 1967 (22.82%) | | 1451 (16.83%) | 560 (13.57%) |  |
| **Prosocial behavior** |  |  |  | |  |  | < .001 |
| <6 | 10558 (49.41%) |  | 4450 (51.63%) | | 4247 (49.27%) | 1861 (45.08%) |  |
| ≥6 | 10808 (50.59%) |  | 4169 (48.37%) | | 4372 (50.73%) | 2267 (54.92%) |  |

Note: Continuous variables are expressed as mean±SD. Categorical variables are expressed as frequency (n,%).

Table S3. Unadjusted association between variables and total difficulties and prosocial behavior problems (N=21,366)

|  | **Statistics** | **Total difficulties** | **Prosocial behavior** |
| --- | --- | --- | --- |
| **Child gender** |  |  |  |
| Boys | 11062 (51.77%) | 1 (ref) | 1 (ref) |
| Girls | 10304 (48.23%) | 0.88 (0.82, 0.94) .0002 | 1.20 (1.14, 1.27) < .001 |
| **Child age, years** | 4.82 ± 0.89 | 0.98 (0.94, 1.02) .2561 | 1.22 (1.19, 1.26) < .001 |
| **Primary parent gender** |  |  |  |
| Male | 5108 (23.91%) | 1 (ref) | 1 (ref) |
| Female | 16258 (76.09%) | 0.73 (0.67, 0.79) < .001 | 1.32 (1.24, 1.40) < .001 |
| **Primary parent age, years** | 34.75 ± 4.56 | 0.98 (0.98, 0.99) < .001 | 1.01 (1.00, 1.02) .0010 |
| **CES-D** | 11.20 ± 6.62 | 1.11 (1.10, 1.12) < .001 | 0.95 (0.94, 0.95) < .001 |
| **Annual family income** |  |  |  |
| Low-income | 8619 (40.34%) | 1 (ref) | 1 (ref) |
| Middle- income | 8619 (40.34%) | 0.68 (0.63, 0.74) < .001 | 1.10 (1.04, 1.17) .0020 |
| High-income | 4128 (19.32%) | 0.53 (0.48, 0.59) < .001 | 1.30 (1.21, 1.40) < .001 |
| **Number of children** |  |  |  |
| 1 | 6148 (28.77%) | 1 (ref) | 1 (ref) |
| 2 | 12844 (60.11%) | 0.94 (0.87, 1.02) .1165 | 0.96 (0.90, 1.02) .1594 |
| ≥ 3 | 2374 (11.11%) | 1.12 (1.00, 1.26) .0564 | 0.98 (0.89, 1.08) .6530 |
| **Education level** |  |  |  |
| ≤ Junior high school | 5741 (26.87%) | 1 (ref) | 1 (ref) |
| High school diploma | 4093 (19.16%) | 0.68 (0.62, 0.75) < .001 | 1.17 (1.08, 1.26) .0002 |
| junior college | 5383 (25.19%) | 0.65 (0.59, 0.71) < .001 | 1.12 (1.04, 1.20) .0033 |
| ≥ Undergraduate degree | 6149 (28.78%) | 0.53 (0.48, 0.58) < .001 | 1.22 (1.13, 1.31) < .001 |
| **Occupation** | 2754 (16.94%) | 0.78 (0.72, 0.85) < .001 | 0.78 (0.72, 0.85) < .001 |
| Education/medical workers | 2917 (13.65%) | 1 (ref) | 1 (ref) |
| Enterprise managers/government civil servants/technicians | 3866 (18.09%) | 1.15 (1.01, 1.32) .0372 | 0.84 (0.76, 0.92) .0003 |
| Sales staff/service workers /freelancers | 5455 (25.53%) | 1.26 (1.11, 1.42) .0003 | 0.86 (0.79, 0.94) .0013 |
| housewife/husband | 5218 (24.42%) | 1.56 (1.38, 1.76) < .001 | 0.83 (0.76, 0.91) < .001 |
| Unemployment/retirement/others | 3910 (18.30%) | 1.55 (1.36, 1.76) < .001 | 0.75 (0.68, 0.83) < .001 |
| **Marital status** |  |  |  |
| Married/cohabitating | 20761 (97.17%) | 1 (ref) | 1 (ref) |
| Widowed/divorced/separated | 605 (2.83%) | 1.54 (1.28, 1.85) < .001 | 1.15 (0.98, 1.36) .0840 |
| **Smoking status** |  |  |  |
| No | 18187 (85.12%) | 1 (ref) | 1 (ref) |
| Yes | 3179 (14.88%) | 1.41 (1.29, 1.54) < .001 | 0.76 (0.71, 0.82) < .001 |
| **Alcohol status** |  |  |  |
| No | 17858 (83.58%) | 1 (ref) | 1 (ref) |
| Yes | 3508 (16.42%) | 1.47 (1.35, 1.60) < .001 | 0.74 (0.69, 0.80) < .001 |
| **Household registration of children** |  |  |  |
| Urban | 7116 (33.31%) | 1 (ref) | 1 (ref) |
| rural | 14250 (66.69%) | 1.34 (1.24, 1.44) < .001 | 0.93 (0.88, 0.99) .0132 |
| **Attended early education program** |  |  |  |
| Yes | 6847 (32.05%) | 1 (ref) | 1 (ref) |
| No | 14519 (67.95%) | 1.02 (0.95, 1.10) .6298 | 0.88 (0.83, 0.93) < .001 |

Table S4 (Supplementary data for Table 5) Mediation effects of parental depressive symptoms on the association between family income and total difficulties (N=21,366)

| **Comparison** | **Effect** | **Estimate** | **95% CI** | ***p*-value** |
| --- | --- | --- | --- | --- |
| Middle- vs. Low-income | Path a (X → M) | -0.9998 | [-1.1958, -0.8065] | < .001 |
|  | Path b (M → Y) | 0.0968 | [0.0906, 0.1031] | < .001 |
|  | Path c' (Direct effect) | -0.2371 | [-0.3169, -0.1591] | < .001 |
|  | Indirect effect (a × b) | -0.0968 | [-0.1164, -0.0775] | < .001 |
|  | Total effect | -0.3179 | [-0.3907, -0.2441] | < .001 |
|  | Proportion mediated | 30.46% | [23.56%, 40.24%] | < .001 |
| High- vs. Low-income | Path a (X → M) | -2.0898 | [-2.3498, -1.8259] | < .001 |
|  | Path b (M → Y) | 0.1020 | [0.0940, 0.1100] | < .001 |
|  | Path c' (Direct effect) | -0.2907 | [-0.4119, -0.1767] | < .001 |
|  | Indirect effect (a × b) | -0.2131 | [-0.2453, -0.1824] | < .001 |
|  | Total effect | -0.4809 | [-0.5990, -0.3692] | < .001 |
|  | Proportion mediated | 44.32% | [36.09%, 58.30%] | < .001 |

Table S5 (Supplementary data for Table 5) Mediation effects of parental depressive symptoms on the association between family income and prosocial behavior (N=21,366)

| **Comparison** | **Effect** | **Estimate** | **95% CI** | ***p*-value** |
| --- | --- | --- | --- | --- |
| Middle- vs. Low-income | Path a (X → M) | -0.9998 | [-1.1958, -0.8065] | < .001 |
|  | Path b (M → Y) | -0.0501 | [-0.0618, -0.0384] | < .001 |
|  | Path c' (Direct effect) | 0.0410 | [-0.0198, 0.1079] | .2108 |
|  | Indirect effect (a × b) | 0.0501 | [0.0401, 0.0610] | < .001 |
|  | Total effect | 0.0876 | [0.0255, 0.1506] | .0064 |
|  | Proportion mediated | 57.16% | [32.39%, 85.16%] | .0020 |
| High- vs. Low-income | Path a (X → M) | -2.0898 | [-2.3498, -1.8259] | < .001 |
|  | Path b (M → Y) | -0.0546 | [-0.0669, -0.0423] | < .001 |
|  | Path c' (Direct effect) | 0.1545 | [0.0710, 0.2406] | .0003 |
|  | Indirect effect (a × b) | 0.1142 | [0.0969, 0.1354] | < .001 |
|  | Total effect | 0.2607 | [0.1815, 0.3451] | < .001 |
|  | Proportion mediated | 43.80% | [31.66%, 67.49%] | < .001 |

Table S6 Association between family income and child mental health outcomes using multiple imputation (pooled estimates from 5 imputed datasets)

|  | Model1 | |  | Model2 | |  |  | Model 3 | |
| --- | --- | --- | --- | --- | --- | --- | --- | --- | --- |
|  | OR (95% CI) | *p* value |  | OR (95% CI) | *p* value |  |  | OR (95% CI) | *p* value |
| **Total difficulties** |  |  |  |  |  |  |  |  |  |
| Low-income | 1.0 (ref) |  |  | 1.0 (ref) |  |  |  | 1.0 (ref) |  |
| Middle-income | 0.64 [0.60, 0.69] | < .001 |  | 0.64 [0.60,0.69] | < .001 |  |  | 0.69 [0.64,0.74] | < .001 |
| High-income | 0.51 [0.46, 0.56] | < .001 |  | 0.50 [0.46, 0.55] | < .001 |  |  | 0.57 [0.51, 0.63] | < .001 |
| **Prosocial behavior** | |  |  |  |  |  |  |  |  |
| Low-income | 1.0 (ref) |  |  | 1.0 (ref) |  |  |  | 1.0 (ref) |  |
| Middle-income | 1.13 [1.07, 1.19] | < .001 |  | 1.14 [1.08, 1.21] | < .001 |  |  | 1.13 [1.07, 1.19] | < .001 |
| High-income | 1.31 [1.22, 1.41] | < .001 |  | 1.36 [1.27, 1.46] | < .001 |  |  | 1.33 [1.23, 1.43] | < .001 |

Note: Model 1: Crude model without adjustment.

Model 2: Adjusted for child gender, child age, primary parent gender, primary parent age.

Model 3: Model 2 plus number of children, smoke, alcohol use,marital status, education level, occupation, household registration of children and early education.
